# Multi-stage structure-based virtual screening approach combining 3D pharmacophore, docking and molecular dynamic simulation towards the identification of potential selective PARP-1 inhibitors

**DOI:** 10.1186/s13065-025-01389-2

**Published:** 2025-02-01

**Authors:** Mahmoud A. El Hassab, Wagdy M. Eldehna, Ghaneya S. Hassan, Sahar M. Abou-Seri

**Affiliations:** 1 Department of Medicinal Chemistry, Faculty of Pharmacy, King Salman International University (KSIU), South Sinai, Ras Sudr, 46612 Egypt; 2 Department of Pharmaceutical Chemistry, Faculty of Pharmacy, Kafrelsheikh University, P.O. Box 33516, Kafrelsheikh, Egypt; 3 Department of Pharmaceutical Chemistry, Faculty of Pharmacy, Pharos University in Alexandria, Canal El Mahmoudia St, Alexandria, 21648 Egypt; 4 Pharmaceutical Chemistry Department, Faculty of Pharmacy, Cairo University, Cairo, 11562 Egypt; 5 Pharmaceutical Chemistry Department, School of Pharmacy, Badr University in Cairo (BUC), Badr City, Cairo 11829 Egypt

**Keywords:** Phthalimide, Selective PARP-1 inhibitor, Structure-based virtual screening, Pharmacophore, Molecular dynamics

## Abstract

Presently, humanity is confronted with a range of diseases that have high death rates, especially those linked to cancerous growths. Several enzymes and proteins have been discovered as highly attractive targets for cancer treatment. The PARP family consists of 17 members and plays a crucial role in repairing DNA damage, which enables the survival of cancer cells. PARP-1 and, to a lesser extent, PARP-2 display above 90% activity in response to DNA damage, thereby distinguishing them apart from other members of the PARP family. Elevated levels of PARP-1 were observed in many types of tumor cells, such as breast, lung, ovarian, prostate, and melanomas. In an attempt to provide a future guide for developing selective inhibitors for PARP-1 over PARP-2 to minimize the resulting side effects from PARP-2 inhibitors, we constructed a structure-based virtual screening approach (SBVS). Firstly. A 3D pharmacophore was constructed based on the interaction of the selective inhibitor compound **IV**. After that, a database of nearly 450,000 phthalimide-containing inhibitors was screened through the validated pharmacophore, and 165 compounds were retrieved. The retrieved compounds were docked into the active site of PARP-1 where only 5 compounds **MWGS-1-5** achieved a favorable docking score than the reference **IV** (-16.8 Kcal/mol). Redocking of the five compounds should have excellent selectivity for PARP-1 over PARP-2, especially compound **MWGS-1**. Further endorsement via molecular dynamics has proven higher affinity and selectivity for **MWGS-1** towards PARP-1 over PARP-2, in which PARP-1- **MWGS-1 and** PARP-1- **MWGS-1** achieved RMSD values of 1.42 and 2.8 Å, respectively.

**Supplementary Information:**

The online version contains supplementary material available at 10.1186/s13065-025-01389-2.

## Introduction

Cancer is a leading cause of death worldwide, characterized by fast and uncontrolled cell development [1]. Early treatments were largely concerned with triggering cell death in quickly dividing cells, particularly by interrupting DNA synthesis and replication [2]. However, due to their mechanism and the development of resistance, previous chemotherapy techniques frequently resulted in considerable adverse effects [2]. As a result, there is an urgent need for more effective and safer anticancer medications that target cancer cells while preserving healthy cells. Current treatments, known as targeted therapies, seek to identify and attack biomarkers particular to cancer cells, such as altered or overexpressed proteins [3].

Poly(ADP-ribose) polymerases (PARPs) are a family of nuclear enzymes found in eukaryotes that includes at least 17 members [4]. These enzymes use nicotinamide adenine dinucleotide (NAD^+^) to covalently link PAR chains to target proteins, a critical step in DNA damage repair and other biological processes [5]. Notably, PARP-1 and, to a lesser extent, PARP-2 show more than 90% activity in response to DNA damage, separating them from other PARP family members [6, 7]. PARP1 has a DNA binding domain that allows it to interact with damaged DNA, therefore engaging in the base excision repair (BER) pathway [8].

**Fig. 1:**
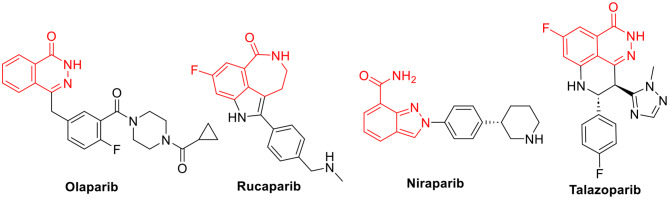
Clinically approved PARP-1 inhibitors

PARP1 levels are elevated in a variety of tumor cells, including breast, lung, ovarian, prostate, and melanomas. As a result, blocking PARP1 is a promising method for treating a wide range of tumors [9]. Several PARP inhibitors have been developed, either as selective monotherapy or in conjunction with other drugs, to treat various cancer types [10]. Olaparib (AZD2281), Rucaparib (AG014699), Niraparib (MK-4827), and Talazoparib (BMN-673) are some of the approved PARP1 inhibitors [9, 11–13] (Fig. 1).

**Fig. 2:**
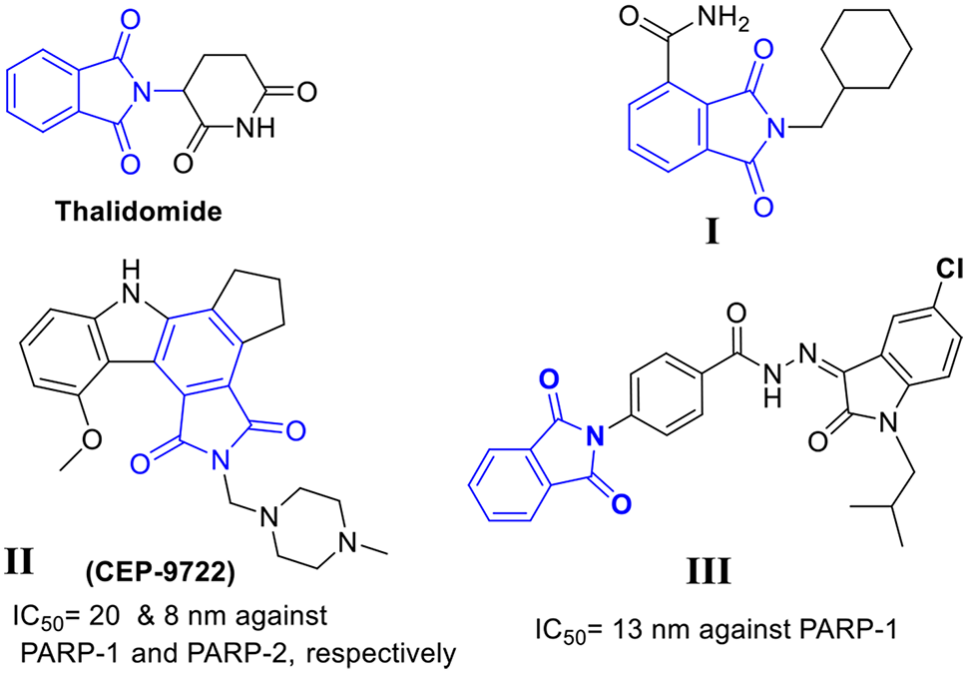
Different isoindoline-1,3-dione (phthalimide) derivatives as antitumor and PARP-1 inhibitors

Different isoindoline-1,3-dione (phthalimide) derivatives highly active against several tumors, such as thalidomide, have been reported (Fig. 2) [14]. In addition, some studies have developed phthalimide-bearing PARP-1 inhibitors, such as compound **I**, CEP-9722 (**II**), and compound **III** (Fig. 2) [15–17]. Recently, our group developed a series of potent phthalimide-based inhibitors, in which **III** showed IC_50_ of 13 nm against PARP-1, respectively [17]. Although this study proved the importance of phthalimide for PARP-1 inhibition, the effect of phthalimide on selectivity was not explored. Accordingly, in this study, we aimed to explore the potential role of phthalimide in the selective inhibition of PARP-1.

Although many studies have successfully reported various potent PARP-1 inhibitors, the selectivity of those inhibitors is still a major issue. The major plethora of the discovered inhibitors demonstrates similar inhibitory profiles against both PARP-1 and PARP-2 [18]. Despite the clinical success of PARP-1/2 inhibitors, these drugs have exhibited notable toxicities, primarily hematological toxicities such as anemia, neutropenia, and thrombocytopenia [19]. These effects seem to be prevalent among all drugs in this class, indicating that their toxicity is linked to their main pharmacological properties [20]. Studies utilizing knockout mice have revealed an intriguing connection between PARP-2 and hematological toxicity [21]. Furthermore, it has been observed that the simultaneous knockout of PARP-1 and PARP-2 is embryonically lethal [22]. Additionally, research conducted by Murai et al. and Ronson et al. has shown that synthetic lethality with BRCA mutations is only caused by PARP-1, in other words, the entrapment of PARP-2 to DNA is not necessary for synthetic lethality [23, 24].

**Fig. 3:**
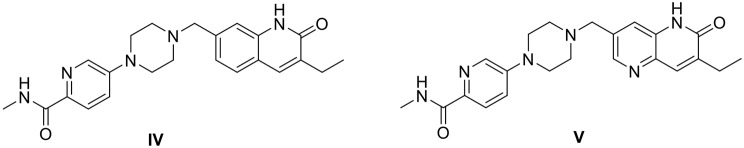
The structure of two selective PARP-1 inhibitors [25]

This created the urge to find new PARP-1 inhibitors with superior selectivity for PARP-1 over PARP-2. To the best of our knowledge, few studies have successfully reported the development of selective PARP-1 inhibitor. Johannes et al., in 2021, reported the discovery of compound **IV** and AZD5305 (**V**), as novel potent and selective PARP1 inhibitors and PARP1–DNA trappers with excellent in vivo efficacy in a BRCA mutant HBCx-17 PDX model [25]. Compound IV showed excellent inhibitory and selectivity profiles with IC_50_ 3 nm and 1400 nm against PARP-1 and PARP-2, respectively [25] (Fig. 3).

**Fig. 4:**
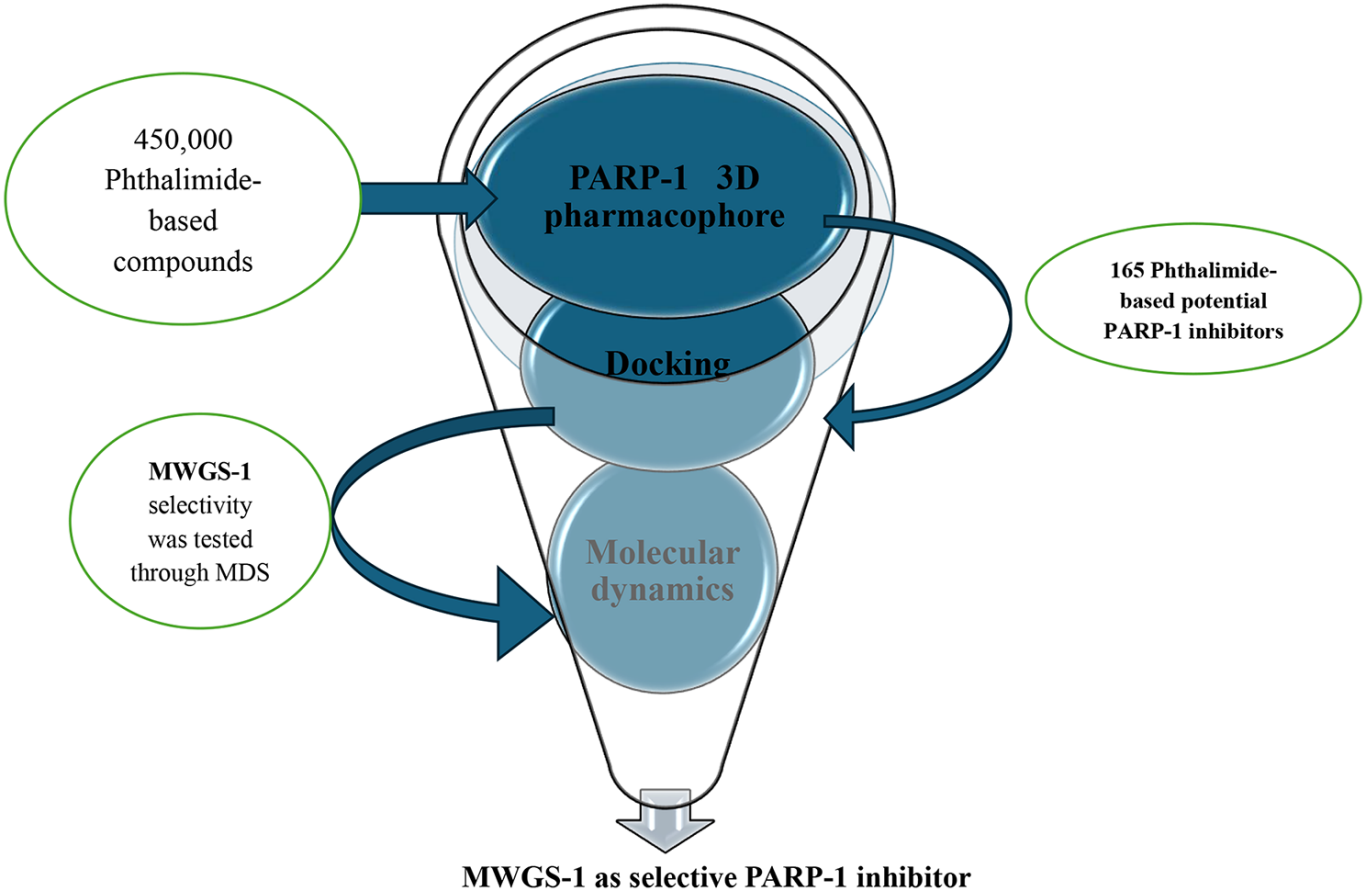
The constructed SBVS approach consists of three stages: 3D pharmacophore generation and mapping, docking, and molecular dynamics

Adding to this, molecular modeling and structure-based-drug design (SBDD) strategies have gained a lot of interest in speeding up the drug discovery process. To this end, our team aimed to develop a structure-based virtual screening (SBVS) approach to give insights into developing selective PARP-1 inhibitors (Fig. 4). Firstly, SBVS started with the generation of a seven-featured pharmacophore based on the essential interactions of compound **IV** with the PARP-1 active site. After that, a database of phthalimide-containing compounds retrieved from PubChem was filtered through the Pharmacophore to find potential selective PARP-1 inhibitors. Following this, a docking study of the filtered phthalimides into the active site of both PARP-1 and PARP-2 was performed to find selective inhibitors. Finally, molecular dynamic simulations were conducted to verify and endorse the entire virtual screening approach. In conclusion, the constructed SBVS approach successfully proved its efficiency in differentiating between selective and nonselective PARP-1 inhibitors and resulted in the identification of MWGS-1 as a potential selective PARP-1 inhibitor.

## Materials and methods

### 3D pharmacophore and database generation

The X-ray crystal structure of PARP-1 in complex with a selective inhibitor compound **IV** was downloaded from the protein data bank PDB ID: 7ONT. Based on compound **IV** essential interactions, a 3D pharmacophore was generated by Pharmit web server (https://pharmit.csb.pitt.edu/) that elucidates pharmacophore queries from receptor-ligand structures. Features supported by Pharmit include hydrogen bond acceptors and donors, negative and positive charges, aromatics, and hydrophobic features. The server identifies which of these features are relevant to the protein-ligand interaction using distance cutoffs between corresponding features on the receptor and ligand where only the interacting features are enabled [26]. The generated pharmacophore was tested to exclude nonselective PARP-1 inhibitors through a set of decoys. A database of all the reported phthalimide-based compounds on the PubChem were downloaded, created on Pharmit server, and screened against the generated pharmacophore. The successful compounds in passing the pharmacophore were validated for their ability to bind PARP-1 and PARP-2 by molecular docking.

### Docking of selected phthalimide-based inhibitors

The compounds that successfully passed the pharmacophore filter were moved to the next step of docking. 165 compounds and the X-ray reference (compound **IV**) were docked in the active site of PARP-1 using PDB ID: 7ONT. All compounds with docking scores higher than compound **IV** were docked in the active site of PARP-2 to evaluate their potential selectivity using the PDB ID: 4TVJ.

Autodock Vina and M.G.L. tools were implemented in the docking study, while Discovery studio was implemented in the results visualization. Autodock Vina uses amber force-field and a united-atom scoring function. In addition, Vina uses a global optimization algorithm called a gradient-based local search genetic algorithm to predict the binding mode of small molecules to their target [27]. Firstly, all the ligands and proteins were prepared and saved in the pdbqt format using M.G.L. tools. After that, the active sites of both PARP-1 and PARP-2 were determined from the binding of the co-crystalized ligands with the following dimensions 22*22*22 Å in the x, y and z directions. Finally, results analysis was done based on the docking scores and binding interactions retrieved from the 2D interaction diagrams.

### Molecular dynamic simulations of MWGS-1 against PARP-1 and PARP-2

Six molecular dynamic simulations (MDS) were conducted for 200 ns using GROMACS 2023.2 software [28]. The retrieved docking coordinates of the PARP-1 enzyme in-complex with compound **MWGS-1** and compound **IV**, in addition to the apo PARP-1, were used as input structures for the first group of MDS. In addition, the retrieved docking coordinates of the PARP-2 enzyme in-complex with compound **MWGS-1** and Olaparib, in addition to the apo PARP-2, were used as input structures for the second group of MDS. The receptor and ligand topologies were generated by PDB2gmx (embedded in GROMACS) and Acpype server, respectively, both under AMBER force field [29, 30]. The detailed applied conditions and steps are fully described in the supporting information.

## Results and discussion

### 3D pharmacophore and database generation

Several studies have reported the differences in sequence and structure between PARP-1 and PARP-2. These studies concluded that the PARP-1 active site is wider than PARP-2, in addition to some amino acid variations in the active site, such as Glu763 in PARP-1 being replaced with Gln319 in PARP-2 and Asp766 in PARP-1 extending to Glu322 in PARP-2 [31]. Some studies have successfully utilized those differences in the development of selective PARP-1 inhibitors. Among those studies, compounds **IV** and **V** were discovered by Johannes et al. in 2021 and proved a potent selective inhibitory activity against PARP-1 [25]. In this context, a 3D pharmacophore was constructed using PDB ID 7ONT that contains a selective inhibitor of PARP-1 (compounds **IV**).

**Fig. 5:**
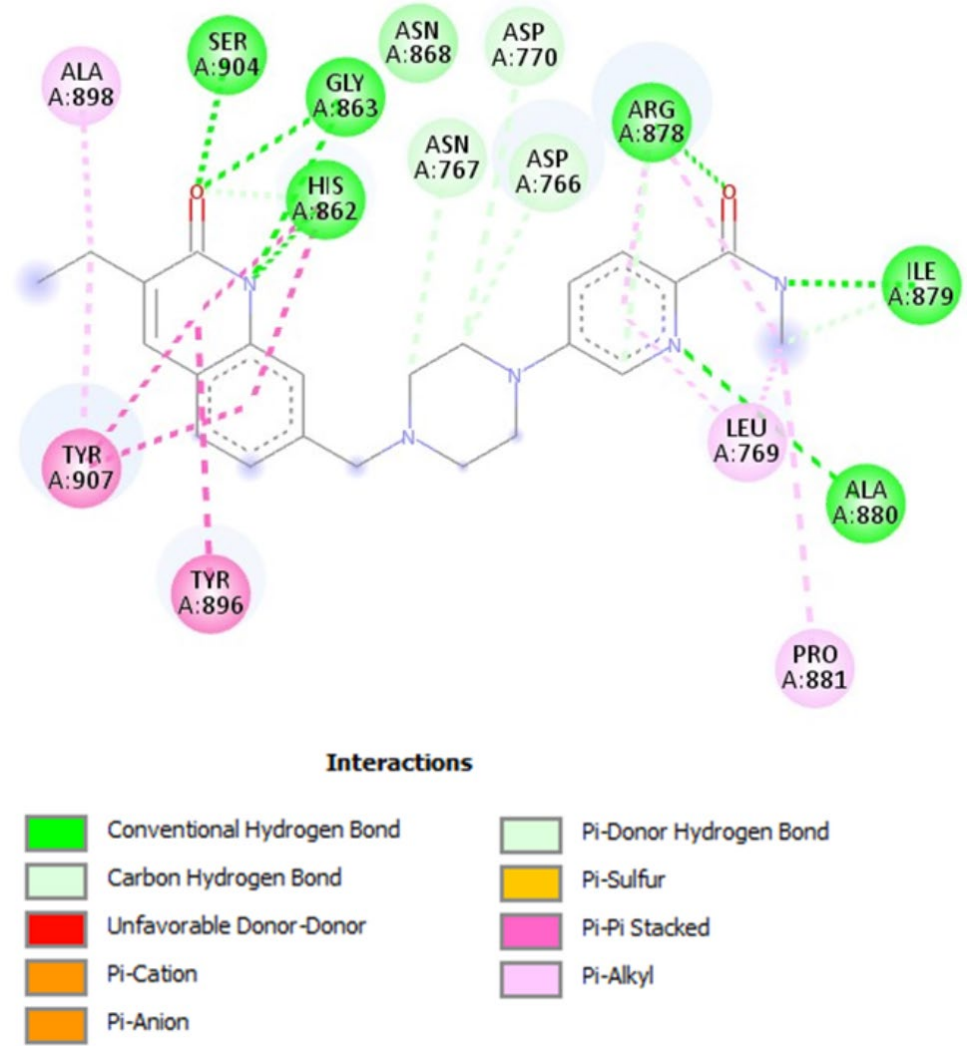
The 2D interaction of compound **IV** with PARP-1 active site

**Fig. 6:**
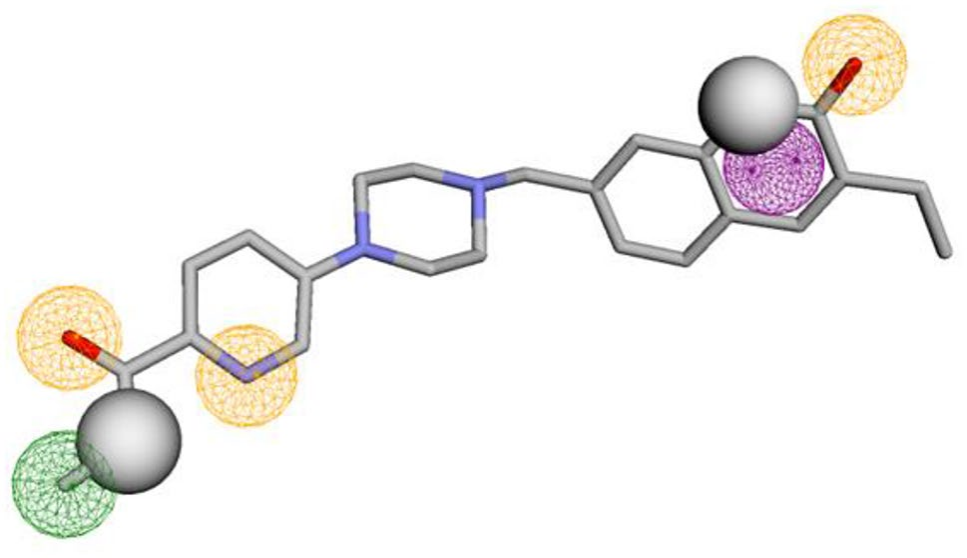
The generated 3D pharmacophore comprises seven features for developing selective PARP-1 inhibitors: the green sphere represents hydrophobic features, the orange sphere represents hydrogen bond acceptor, the grey sphere represents hydrogen bond donor, and the pink sphere represents an aromatic feature

**Fig. 7:**
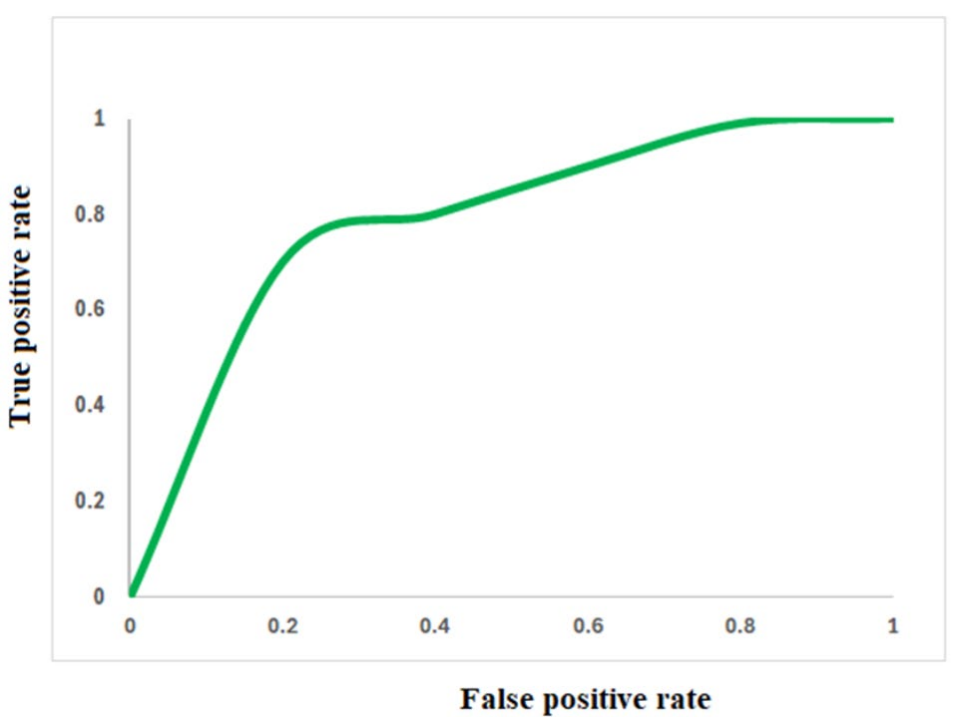
The ROC curve demonstrated an excellent ability to distinguish true positive hits from false results

Accordingly, the Pharmit webserver was implemented to construct the 3D pharmacophore based on the interaction diagram of compounds **IV** Fig. 5. A seven-featured pharmacophore comprising three hydrogen bond acceptors, two hydrogen bond donors, one aromatic and one hydrophobic feature was generated, Fig. 6. The pharmacophore was validated through a database containing 1980 decoy, 7 selective PARP-1 inhibitors, 12 nonselective PARP-1 inhibitors (Supporting information). Interestingly the pharmacophore demonstrated excellent selectivity achieving goodness of hit score and enrichment factor 0.86 and 83, respectively. In addition, the ROC curve demonstrated an excellent ability to distinguish true positive hits from false results Fig. 7.

As we previously highlighted the potential role of phthalimides in targeting PARP-1, a database of all phthalimide derivatives (nearly 450,000 compounds) included in the PubChem was created on the Pharmit server. The phthalimide database was screened using the specified 3D pharmacophore and hits matching all the features were retrieved. This resulted in 165 compounds being moved to the docking stage against PARP-1 enzyme.

### Docking results

**Fig. 8:**
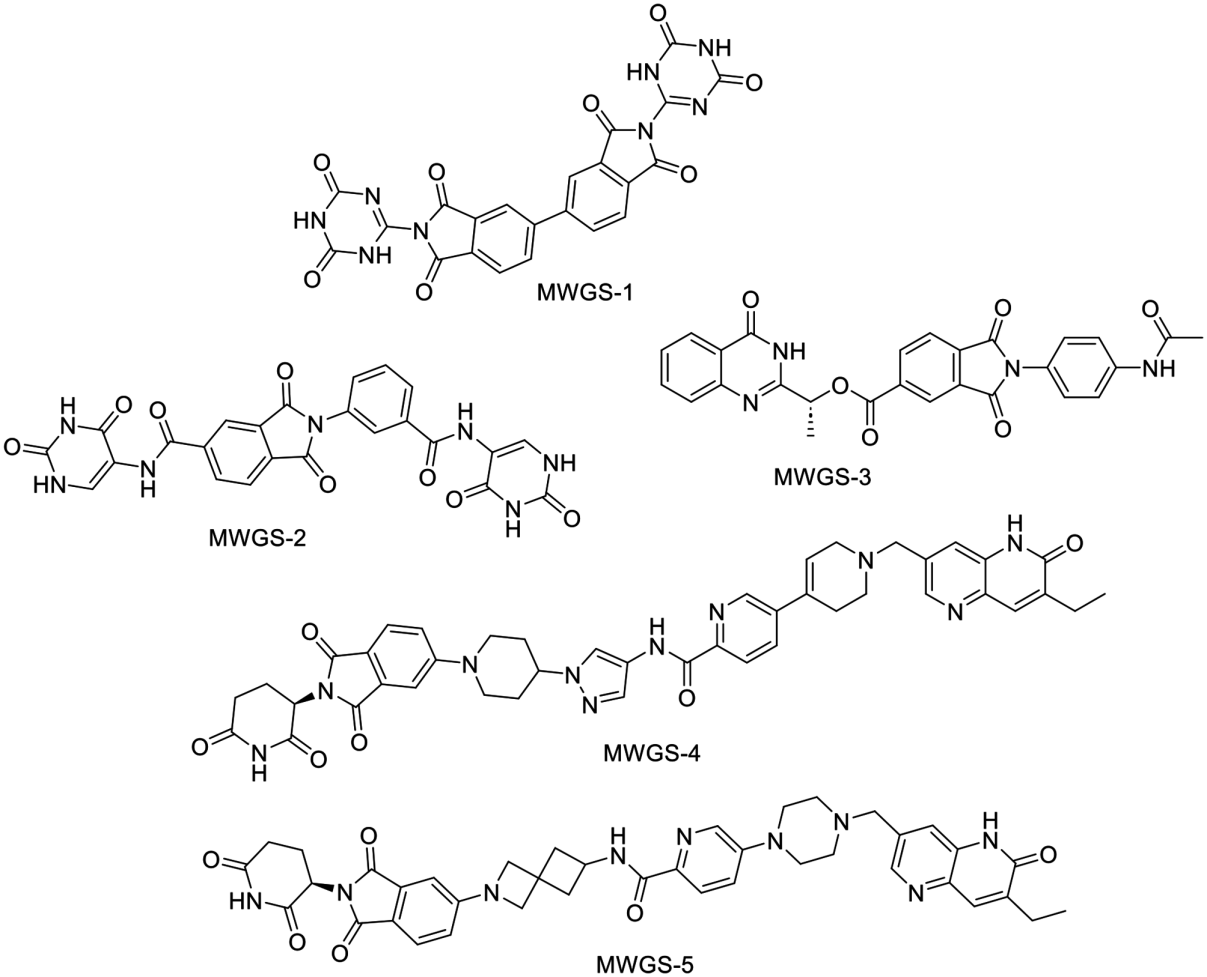
The structure of the best five Phthalimide-based inhibitors achieved the highest docking score against PARP-1

165 compounds successfully passed the pharmacophore filter and were docked into the vicinity of the PARP-1 active site. The crystal reference compound **IV** was redocked in the active site to provide a benchmark value for the 165 compounds. That last mentioned step also resulted in an RMSD value of 0.8 Å between the co-crystalized and redocked poses of compound **IV** Figure S1 supporting information). Amongst the docked 165 compounds, only 5 compounds exceeded the docking score (-16.8 Kcal/mol) of the reference compound **IV** Fig. 8. Those five compounds were selected for further analysis and inspection through the generation of their 2D interaction diagram with PARP-1.

**Fig. 9:**
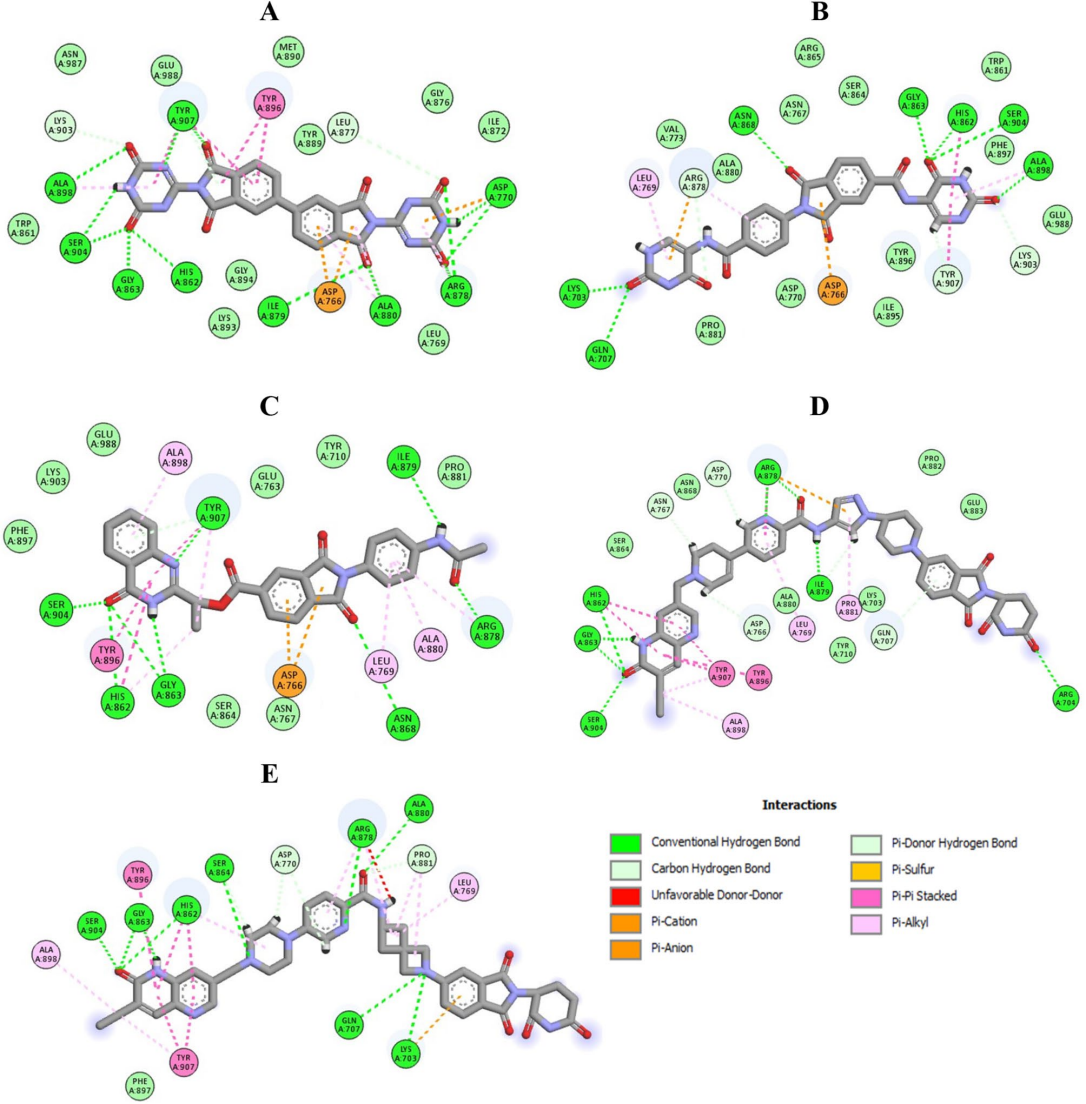
The docking results of PARP-1 with (**A**) **MWGS-1** (**B**) **MWGS-2** (**C**) **MWGS-3** (**D**) **MWGS-4 (E**) **MWGS-5**

Compound **MWGS-1** (Compound CID: 117915228) achieved a docking score of -18.8 Kcal/mol, displaying an excellent interaction mode perfectly aligned with the reported binding pattern of PARP-1 inhibitors. As shown in Fig. 9, **MWGS-1** successfully engaged in hydrogen bond interactions with Asp770, His862, Gly863, Arg878, Ile879, Ala880, Ser904, and Tyr907, in addition it formed hydrophobic interactions with Asp766, Asp770, Leu877, Ala880, Tyr896, Ala898, and Tyr907. Similarly, **MWGS-2** (Compound CID: 4561811) achieved a docking score of – 17.9 Kcal/mol, it established several types of interactions with Lys703, Gln707, Asp766, Leu769, His862, Gly863, Asn868, Arg878, Ala898, Lys903, Ser904, and Tyr907. Moreover, compound **MWGS-3** (Compound CID: 135887765) achieved a docking score of – 17.4 Kcal/mol, it formed several types of interactions with Asp766, Leu769, His862, Gly863, Asn868, Arg878, Ile879, Ala880, Tyr896, Ala898, Ser904, and Tyr907. Finally, compounds **MWGS-4** (Compound CID: 168228806) and **MWGS-5** (Compound CID: 168228781) achieved the same docking score − 16.9 Kcal/mol. As seen in Fig. 8, both compounds had a similar binding mode in which **MWGS-4** interacted with Arg704, Gln707, Asp766, Asn767, Leu769, Asp770, His862, Gly863, Arg878, Pro881, Tyr896, Ala898, Lys903, Ser904, and Tyr907, while compound **MWGS-5** interacted with Lys703, Gln707, Leu769, Asp770, His862, Gly863, Ser864, Arg878, Ala880, Pro881, Tyr896, Ala898, Ser904, and Tyr907. It is worth noting that the five compounds were successfully involved in hydrogen bonding and hydrophobic interactions with the essential residues His862, Gly863, and Ser903. Besides, all the compounds except **MWGS-5** successfully interacted with Asp766, a critical residue for selective PARP-1 inhibitors.

**Fig. 10:**
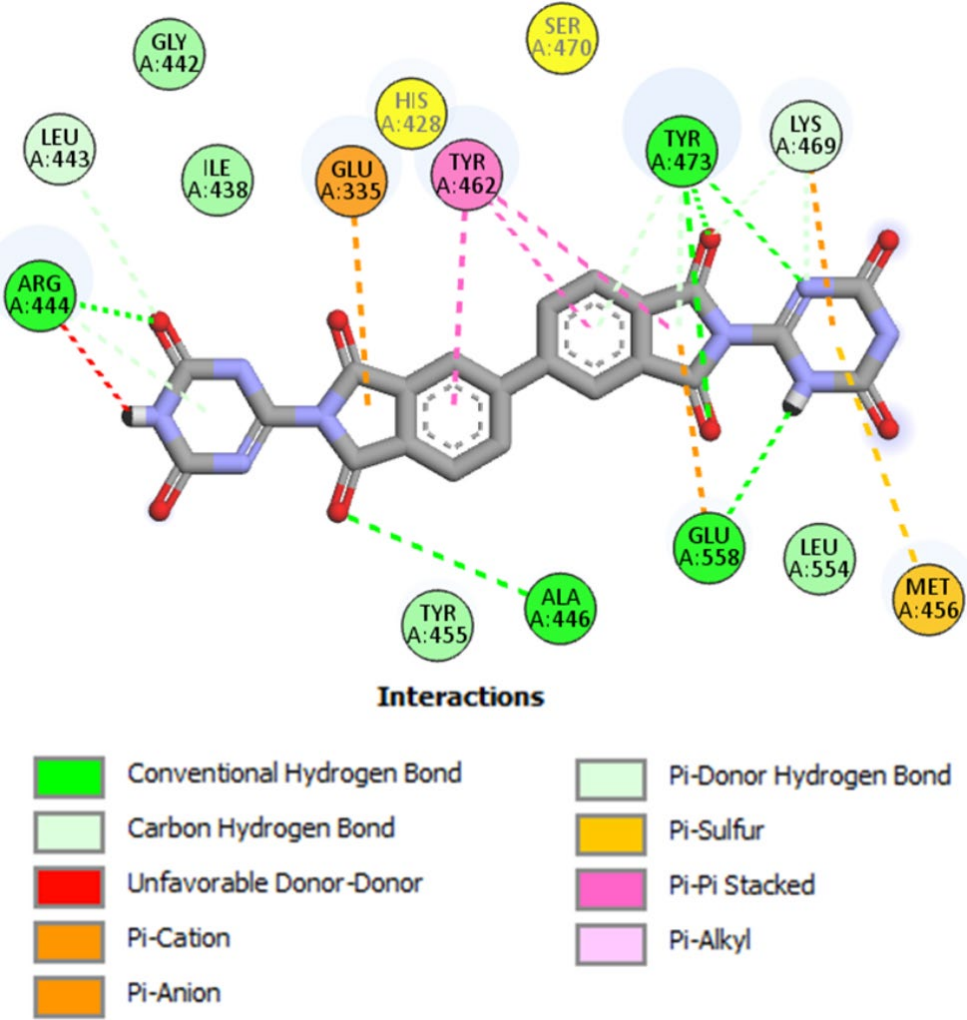
The docking pose of **MWGS-1** within PARP-2 active site

To evaluate the selectivity of the five compounds, they were docked into the active site of PARP-2 using Olaparib as a reference. Interestingly, compounds **MWGS-1**,** MWGS-2**,** MWGS-3, MWGS-4** and **MWGS-5** achieved docking scores of -9.4, -10.4, -9.6, -11.7 and − 16.7, Kcal/mol, respectively, while Olaparib scored − 20.2 Kcal/mol. These results concluded that compounds **MWGS-1-4** may have excellent selectivity for PARP-1 over PARP-2, while compound **MWGS-5** failed to demonstrate any significant selectivity. Inspecting the binding mode of **MWGS-1** with PARP-2, the following was noticed: **MWGS-1** failed to achieve the required interaction pattern with PARP-2. **MWGS-1** missed the interaction with the critical residues His428 and Ser470, two essential residues for PARP-2 inhibition; in addition, **MWGS-1** formed unfavorable donor-donor interaction with Arg444 Fig. 10.

**Fig. 11:**
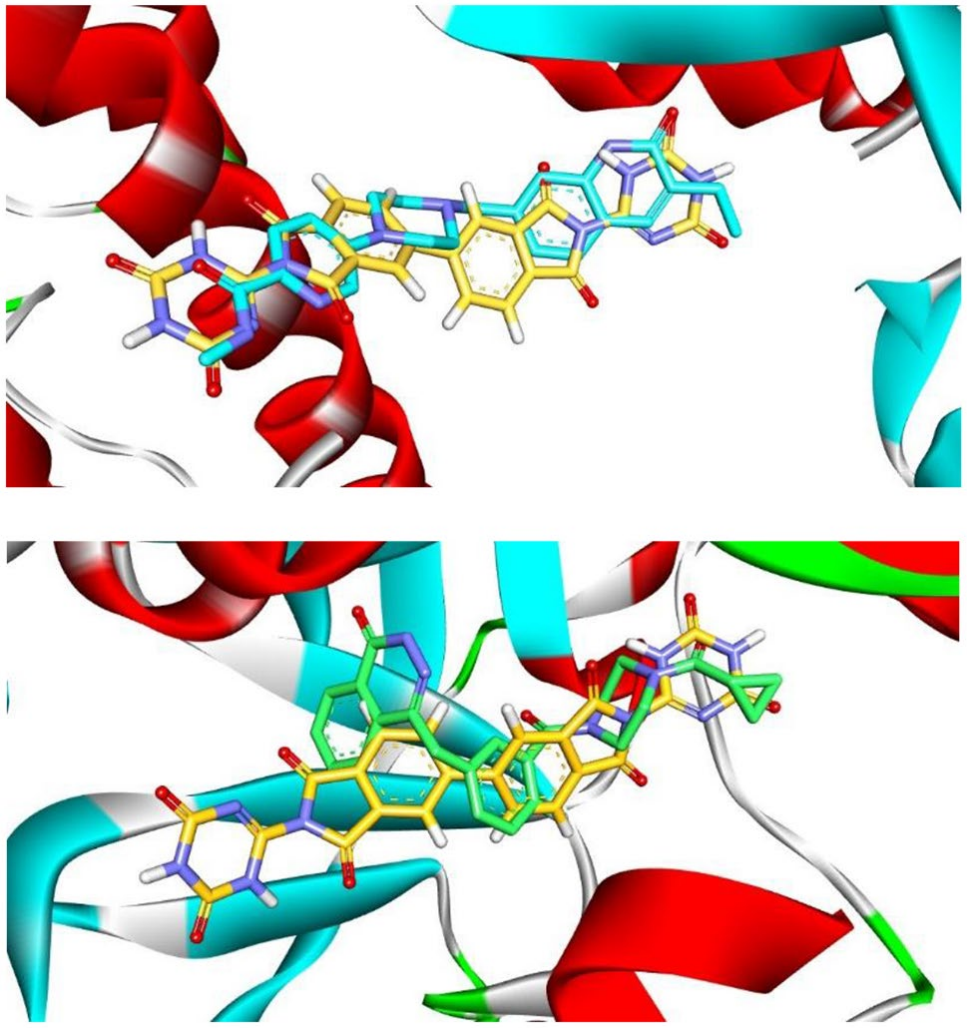
Molecular alignment between **MWGS-1** and compound **IV** within PARP-1 active site showing a perfect overlay of both ligands (left side). The overlay of **MWGS-1** and Olaparib in the binding site of PARP-2 revealed the inability of **MWGS-1** to occupy the same position as Olaparib (right side). **MWGS-1** is colored orange, while compound **IV** is in cyan and Olaparib are in green

To justify the retrieved results from the docking studies, molecular alignment was done between the docked pose of **MWGS-1** with PARP-1 and PARP-2 and the corresponding co-crystalized ligands in each target Fig. 11. Interestingly, the results demonstrated perfect alignment of **MWGS-1** and compound **IV** in the active site vicinity of PARP-1; in contrast, **MWGS-1** had a different orientation than Olaparib in the active site of PARP-2. This finding is thought to be attributed to the rigidity and linearity of **MWGS-1**, which enables its perfect fit in the wider active site of PARP-1, while the same feature hindered its orientation in the narrower active site of PARP-2. In conclusion, the docking results endorsed the ability of the generated pharmacophore to find selective PARP-1 inhibitors and suggested a strong relation between compound rigidity and selectivity.

### Molecular dynamic simulation (MDS) results

#### RMSD analysis

In the current study, further in silico investigations were achieved through molecular dynamic simulations. Molecular dynamics (MD) simulation provides many valuable information and parameters to study the dynamicity of biological systems. Amongst this information, MD could provide insights into precise estimation of the binding strength of a docked complex of a ligand and its target. Accordingly, the predicted binding coordinates retrieved from the docking of PARP-1 and PARP-2 with **MWGS-1**, in addition to PARP-1-**IV** and PARP-2-Olaparib complexes, were moved forward to MD simulation. To provide a comparative mean for the effect of each ligand on the stability of the PARP-1 and PARP-2 enzymes, they were subjected to MDS using the Apo form.

**Fig. 12:**
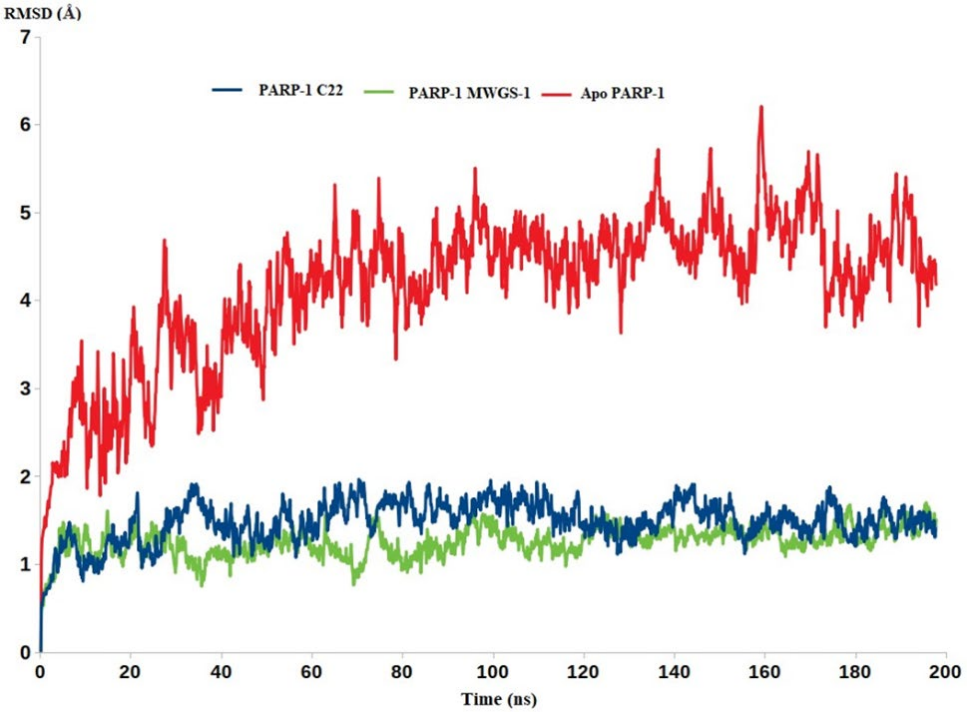
The RMSD of the Apo PARP-1 (red), PARP-1-**IV** (blue), and PARP-**1 MWGS-1**

As demonstrated by Fig. 12, the two inhibitors **MWGS-1** and compound **IV** were able to stabilize the PARP-1 enzyme as indicated by their lower RMSD values compared to the RMSD value of Apo PARP-1. The PARP-1-**IV** complex had an average RMSD value of 1.58Å, that was very close to that of **MWGS-1**-PARP-1 (1.42 Å), while the RMSD of the Apo PARP-1 reached 4.22 Å.

In cancer cells, PARP-1 serves as the DNA repairing machine, increasing the survival of cancer cells, and allowing uncontrolled cell growth. In this respect, the high dynamicity seen in the Apo PARP-1 as discerned from the high RMSD values is perfectly aligned with its intended oncogenic function. The capability of compound **MWGS-1** to restrict the dynamic nature of the PARP-1 via the formation of stable complexes as indicated by the lower RMSD values is a valid indicator for their inhibitory impact on PARP-1 Fig. 12.

**Fig. 13:**
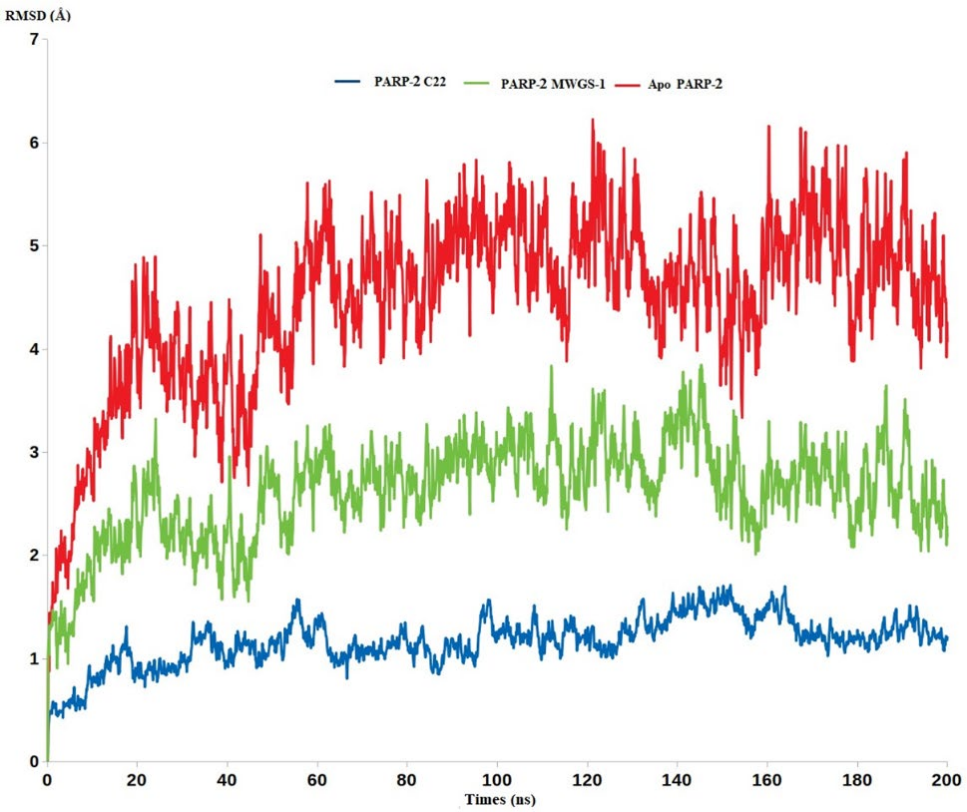
The RMSD of the Apo PARP-2 (red), PARP-2 Olaparib (blue), and PARP-2 **MWGS-1**

Furthermore, another MDS for PARP-2 was conducted for extra validation of the retrieved results. As Fig. 13. reveals PARP-2-Olaparib complex demonstrated the highest stability showing RMSD values less than 1.2 Å. In contrast, PARP-2-**MWGS-1** showed less stability and significant degree of dynamicity having RMSD value of more than 2.8 Å, while the apo PARP-2 showed RMSD value of 5.3 Å.

In conclusion, the MDS results have proven the potential selectivity of **MWGS-1** for PARP-1 over PARP-2 as indicated by the excellent stability shown in **MWGS-1-**PARP-1 unlike the **MWGS-1-**PARP-2 complex that showed significant decrease in the complex stability. Accordingly, the overall steps of virtual screening approach validate each other and give a high credibility to the discovered potential selective inhibitor of PARP-1.

#### Binding free energy calculations using MM-PBSA approach

Attempting to further evaluate the strength of binding between the PARP-1 and PARP-2 enzymes and compound **MWGS-1**, the gmx_mmpbsa package was brought in action to calculate the binding free energies resulting from the binding of **MWGS-1** to the PARP-1 and PARP-2 enzymes [32]. The generated trajectories from the production stage were used to calculate all the forms of binding free energy. These energy types include Electrostatic energy, van der Waal energy, Polar solvation energy and SASA energy. All the previous types of energy were calculated for the two complexes containing PARP-1 and PARP-2 bound to **MWGS-1** Table 1. Interestingly, the calculated binding free energy for the **MWGS-1**-PARP-1 was much more favorable than the calculated binding free energy for the **MWGS-1**-PARP-2. **MWGS-1** achieved binding free energy of − 380.9 ± 4.5 (kJ/mol) with PARP-1, whereas the same compound achieved − 197.2 ± 3.6 (kJ/mol). These results augmented all the in-silico calculations giving credit to the entire virtual screening approach in discovering new selective PARP-1 inhibitors.

**Table 1:** Summary of the interaction energies and the binding free energy for both the complexes

Complex	ΔE_Binding (KJ/mol)_	ΔE_Electrostatic (KJ/mol)_	ΔE_Van der Waal (KJ/mol)_	ΔE_polar solvation (KJ/mol)_	SASA_(kJ/mol)_
**MWGS-1**-PARP-1	−380.9 ± 4.5	−170.2 ± 4.3	−298.2 ± 5.9	130.2 ± 1.9	−42.7 ± 1.1
**MWGS-1**-PARP-2	−197.5 ± 3.6	−68.2 ± 3.6	−185.7 ± 4.5	86.3 ± 2.6	−29.6 ± 1.0

## Conclusions

In an attempt to provide a future guide for developing selective inhibitors for PARP-1 over PARP-2 to minimize the resulting side effects from PARP-2 inhibitors, we constructed a structure-based virtual screening approach (SBVS). Firstly, A 3D pharmacophore was constructed based on the interaction of the selective inhibitor compound **IV**, where a seven feature Pharmacophore was generated. After that, a database of nearly 450,000 phthalimide-containing inhibitors was screened through the validated pharmacophore, and 165 compounds were retrieved. The retrieved compounds were docked non-clustered into the active site of PARP-1 where only 5 compounds **MWGS-1-5** achieved a favorable docking score compared to the reference **IV** (-16.8 Kcal/mol). Redocking of the five compounds showed excellent selectivity for PAR-P-1 over PARP-2, especially compound **MWGS-1**. The previous findings were attributed to the compounds’ rigidity and linearity, which allows them to fit in PARP-1 compared to PARP-2. To provide a clue for this hypothesis, further endorsement *via* molecular dynamics has proven higher affinity and selectivity for **MWGS-1** towards PARP-1 over PARP-2, in which PARP-1- **MWGS-1** and PARP-2-**MWGS-1** achieved RMSD values of 1.42 and 2.8 Å, respectively.

## Future outlook

It is worth noting that the small molecules identified in this study possess structural features that can be associated with Proteolysis Targeting Chimeras (PROTACs). While our investigation focused mainly on their potential as PARP-1 inhibitors, such structural similarity opens interesting possibilities for their mechanisms of action. The bivalent nature of these molecules suggests they could exhibit PROTAC-like activity in addition to direct inhibition. This dual functionality could enhance their efficacy through both enzyme inhibition and protein degradation. Thus, future studies should explore this aspect and investigate whether these molecules can induce PARP-1 degradation alongside their inhibitory effects. Accordingly, these molecules could represent a novel class of PARP-1 targeting agents, combining the benefits of direct inhibition and targeted protein degradation.

## Electronic supplementary material

Below is the link to the electronic supplementary material.

Supplementary Material 1

## Data Availability

Availability of data and materials: All data generated or analysed during this study are included in this published article and its supplementary information files.
