# Supplementary material for: Multi-stage structure-based virtual screening approach combining 3D pharmacophore, docking and molecular dynamic simulation towards the identification of potential selective PARP-1 inhibitors
Source: BMC Chem. 2025 Feb 1;19(1):30. doi: 10.1186/s13065-025-01389-2 (PMC11786381; doi:10.1186/s13065-025-01389-2)
Supplement: Supplementary file 1 — Supplementary Material 1 [file 13065_2025_1389_MOESM1_ESM.docx]

**Multi-stage structure-based virtual screening approach combining 3D Pharmacophore, docking and molecular dynamic simulation towards the identification of potential selective PARP-1 inhibitors**

Mahmoud A. El Hassab^a, *^, Wagdy M. Eldehna^b^, Ghaneya S. Hassan^c,d^, Sahar M. Abou-Seri^d, *^

*^a^ Department of Medicinal Chemistry, Faculty of Pharmacy, King Salman International University (KSIU), South Sinai, Ras Sudr 46612, Egypt*

*^b^ Department of Pharmaceutical Chemistry, Faculty of Pharmacy, Kafrelsheikh University, Kafrelsheikh, P.O. Box 33516, Egypt*

*^c^ Pharmaceutical Chemistry Department, Faculty of Pharmacy, Cairo University, Cairo 11562, Egypt*

*^d^ Pharmaceutical Chemistry Department, School of Pharmacy, Badr University in Cairo (BUC), Badr City, Cairo 11829, Egypt*

**Table of contents**

Molecular dynamics. **2**

Redocking images **4**

Tanle S1. The structure of selective and nonselective PARP-1 inhibitors used in the validation of the 3D pharmacophore. **5**

**Molecular dynamics**

In this work, six molecular dynamic simulations (MDS) were conducted for 200ns.

using GROMACS 2.1.1 software. The two free enzymes and the retrieved docking

coordinates of the same enzymes bound compound MWGS-1, Olaparib and compound IV were

used as input structures for molecular dynamics. The receptor and ligand topologies

were generated by PDB2gmx (embedded in GROMACS) and Acpype server respectively, both under GROMOS96 force field was implemented to generate the ligand topologies using the. After rejoining ligands and receptor topologies to generate six systems, the typical molecular dynamics scheme of GROMACS was applied for all the systems. This include, solvation, neutralization, energy minimization

under GROMOS96 43a1 force field and two stages of equilibration (NVT and NPT).

Finally, unrestricted production stage of 200ns was applied for the six systems with

particle mesh ewald (PME) method implemented to compute the long-range electrostatic

values using 12 Å cut-off and 12 Å Fourier spacing. The stability of the complexes was

judged using RMSD values calculated from the MDS trajectories from the

production step.

MMPBSA calculations

The following equation was implemented to calculate the binding free energies:

ΔG (Binding)  = G (Complex)  − G (Receptor)  − G (Ligand)

Where G (Complex)  is the total free energy of the protein−ligand complex and G (Receptor)  and

G (Ligand)  are the total free energies of the isolated protein and ligand in solvent,

respectively. The total free energy of any of the three mentioned entities (complex,

receptor and ligand) were calculated for all MD trajectories from its molecular mechanics

potential energy plus the energy of the solvation, using the g_mmpbsa package

implemented in the GROMACS software. Individual energies along with the values of

standard deviations were calculated and then summed together to yield the average total

free energy of each component. Finally, to calculate the binding-free energy, the total free

energy of the receptor and the ligand were subtracted from the total free energy of the

complex.


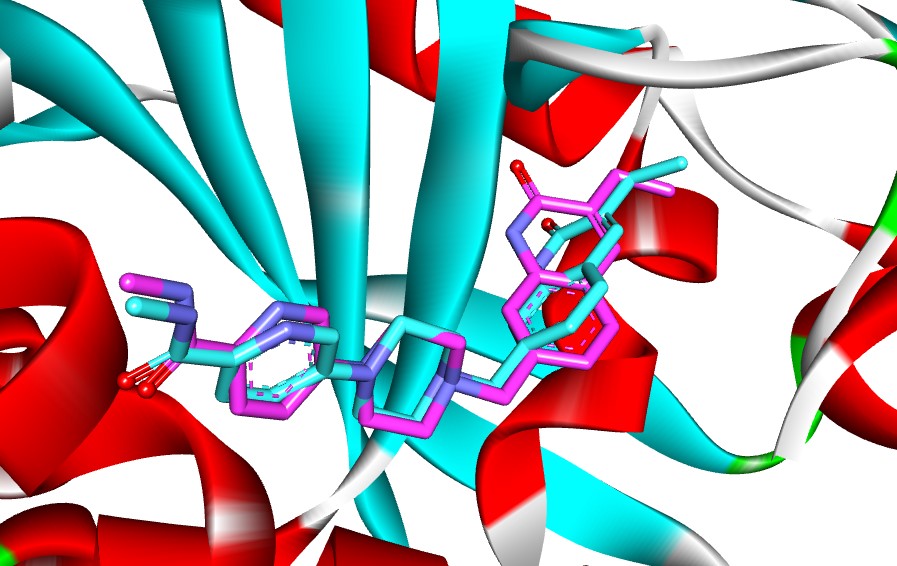


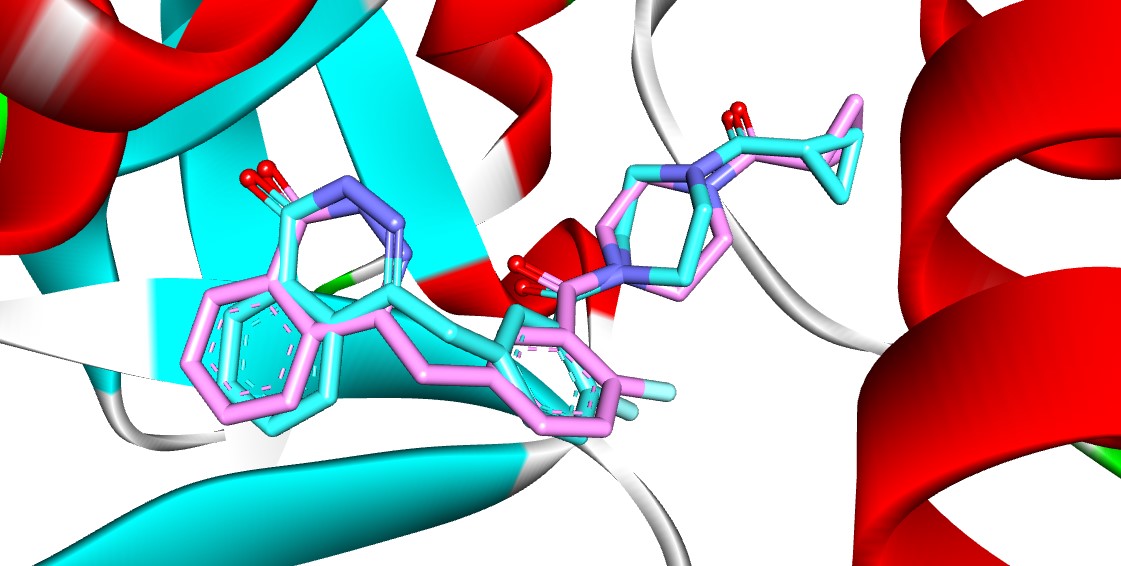


Figure S1. Upper) redocking compound IV in PARP-1 active site Co-crystalized pose in cyan, and redocked pose in pink. Lower) redocking Olaparib in PARP-2 active site, Co-crystalized pose in cyan, and redocked pose in pink

Table s1. The structure of selective and nonselective PARP-1 inhibitors used in the validation of the 3D pharmacophore.

| Compound | Selectivity for PARP-1/PARP-2 | Pharmacophore passing | RMSD | Ref |
| --- | --- | --- | --- | --- |
|   **V** | Selective | Pass | 0.01 | (1) |
|  | Selective | Pass | 0.01 | (1) |
|  | Selective | Pass | 0.01 | (1) |
|  | Selective | Pass | 0.832 | (1) |
|  | Selective | Pass | 0.795 | (1) |
|  | Selective | Fail | --- | (2) |
|   **XI** | Selective | Pass | 0.151 | (1) |
|   **XII** | Non-Selective | pass | 0.632 | (3) |
|  | Non-Selective | Fail | --- | (4) |
|  | Non-Selective | Fail | --- | (5) |
|  | Non-Selective | Fail | --- | (6) |
|  | Non-Selective | Fail | --- | (7) |
|  | Non-Selective | Fail | --- | (2) |
|  | Non-Selective | Fail | --- | (8) |
|  | Non-Selective | Fail | --- | (9) |
|  | Non-Selective | Fail | --- | (9) |
|  | Non-Selective | Fail | --- | (10) |
|  | Non-Selective | Fail | --- | (11) |
|  | Non-Selective | Fail | --- | (11) |
|  | Non-Selective | Fail | --- | (11) |

References

1. JOHANNES, Jeffrey W., et al. Discovery of 5-{4-[(7-ethyl-6-oxo-5, 6-dihydro-1, 5-naphthyridin-3-yl) methyl] piperazin-1-yl}-N-methylpyridine-2-carboxamide (AZD5305): a PARP1–DNA trapper with high selectivity for PARP1 over PARP2 and other PARPs. Journal of Medicinal Chemistry, 2021, 64.19: 14498-14512. ‏
2. *J. Med. Chem.* 2024, 67, 11, 8877–8901
3. Wang, Y., Li, K., Xu, W., & Gou, S. (2023). Design, synthesis, and biological evaluation of a series of benzofuran [3, 2-d] pyrimidine-4 (3H)-one derivatives containing thiosemicarbazone analogs as novel PARP-1 inhibitors. *Bioorganic Chemistry*, *139*, 106759.‏
4. Kayumov, Muzaffar, et al. "Design, synthesis and pharmacological evaluation of new PARP1 inhibitors by merging pharmacophores of olaparib and the natural product alantolactone." *European Journal of Medicinal Chemistry* 240 (2022): 114574.‏
5. Seo, Ju‐Ahn, Jinjae Park, and Cheol‐Hong Cheon. "Synthesis of a Pinacol Boronate Precursor for [18F] Rucaparib Radiosynthesis." *Asian Journal of Organic Chemistry* 13.1 (2024): e202300501.‏
6. <https://ppubs.uspto.gov/pubwebapp/external.html?q=(11364241).pn.&db=USPAT,US-PGPUB>
7. [Deng, Kai](javascript:); [Li, Qiongqiong](javascript:); [Lu, Lina](javascript:); [Wang, Luting](javascript:); [Cheng, Zhiyong](javascript:); [Wang, Suyun](javascript:) [**Biochemistry and Biophysics Reports**, **2024**, vol. 39, art. no. 101781]
8. [Li, Xinxin](javascript:); [Wang, Cheng](javascript:); [Li, Shang](javascript:); [Yin, Fucheng](javascript:); [Luo, Heng](javascript:); [Zhang, Yonglei](javascript:); [Luo, Zhongwen](javascript:); [(…)](javascript:) [Kong, Lingyi](javascript:); [Wang, Xiaobing](javascript:) [**European Journal of Medicinal Chemistry**, **2024**, vol. 265, art. no. 116054]
9. <https://worldwide.espacenet.com/patent/search?q=pn%3DJP2024528725A>
10. [Thakur, Amandeep](javascript:); [Rana, Mandeep](javascript:); [Ritika](javascript:); [Mathew, Jacob](javascript:); [Nepali, Sanya](javascript:); [Pan, Chun-Hsu](javascript:); [Liou, Jing Ping](javascript:); [Nepali, Kunal](javascript:) [**Bioorganic Chemistry**, **2023**, vol. 141, art. no. 106893]
11. [Yang, Fei-Fei](javascript:); [Zhao, Tian-Tian](javascript:); [Milaneh, Slieman](javascript:); [Zhang, Chun](javascript:); [Xiang, Da-Jun](javascript:); [Wang, Wen-Long](javascript:) [**RSC Medicinal Chemistry**, **2024**, vol. 15, # 6, p. 1828 - 1848]
